# Meeting the Demand for Results and Accountability: A Call for Action on Health Data from Eight Global Health Agencies

**DOI:** 10.1371/journal.pmed.1000223

**Published:** 2010-01-26

**Authors:** Margaret Chan, Michel Kazatchkine, Julian Lob-Levyt, Thoraya Obaid, Julian Schweizer, Michel Sidibe, Ann Veneman, Tadataka Yamada

**Affiliations:** 1 World Health Organization, Geneva, Switzerland; 2 Global Fund to Fight AIDS, Tuberculosis and Malaria, Geneva, Switzerland; 3 Global Alliance for Vaccines and Immunisation (GAVI), Geneva, Switzerland; 4 United Nations Population Fund (UNFPA), New York, New York, United States of America; 5 Human Development Network, World Bank, Washington, D.C., United States of America; 6 Joint United Nations Programme on HIV/AIDS (UNAIDS), Geneva, Switzerland; 7 United Nations Children's Fund (UNICEF), New York, New York, United States of America; 8 Global Health Program, Bill & Melinda Gates Foundation, Seattle, Washington, United States of America

## Abstract

Margaret Chan, Director-General of the WHO, and the heads of seven other global health agencies, call for a concerted global effort to collect better health data.

Recent substantial increases in international attention to health have been accompanied by demands for statistics that accurately track health progress and performance, evaluate the impact of health programs and policies, and increase accountability at country and global levels. The use of results-based financing mechanisms by major global donors has created further demand for timely and reliable data for decision-making. In addition, there is increasing country demand for data in the context of health sector strategic plans, including in countries that have established International Health Partnership (IHP+) compacts [1]. In spite of recognized efforts by programs and countries, the ability to respond to this demand is constrained by limited data availability, quality, and use. Many developing countries have limitations that hamper the production of data of sufficient quality and timeliness to permit regular tracking of progress made in scaling up and strengthening health systems. Data gaps span across the range of input, output, outcome, and impact indicators. New ways of working and a more systematic approach by all partners are needed to better monitor and evaluate progress and performance. We believe that this global public good is a necessary foundation to improve health investments and programs and accelerate progress towards the Millennium Development Goals (MDGs) and other major international health goals.

Monitoring and evaluation (M&E) in health requires different types of data, including levels and distribution of health financing, health workforce, service access and quality, intervention coverage, risk factors, and health status, which are derived from multiple sources. Table 1 summarizes the current situation and required actions in developing countries for the main data sources: household surveys, birth and death registration, census, health facility reporting systems including surveillance systems, and administrative data. To improve data availability, quality, and use, each of these data sources need to be strengthened according to international principles and standards, including the Health Metrics Network framework for country health information systems [2],[3]. In this process, strengthening country capacity in collecting, processing, analyzing, and using health data is essential. There are many initiatives to support capacity building, but the current situation tends to be fragmented, often driven by the needs of single-disease programs. Long-term systematic efforts to build the capacities of country institutions are few and far between. Such an approach should promote quasi-autonomous or independent country institutions, which work very closely with ministries of health and national statistical offices. Adherence to the Fundamental Principles of Official Statistics is critical to increase accountability, transparency, and adherence to quality standards [4].

**Table 1:** Health data sources: Situation in countries and required actions.

Data Source	Situation	Required Actions
Surveys	In low- and middle-income countries, household health surveys are the main source of data for monitoring progress towards MDGs (and beyond) including health outcomes, risk factors, coverage, and equity.In spite of progress in harmonization and frequency of international survey programs including DHS, MICS, and some special disease surveys (e.g., HIV, TB, malaria), there is still a need to enhance the availability of comparable data across countries and over time.	• Support development of well-coordinated 10 year national health survey plan, linked to the national health sector plan.• Promote development and implementation of country health-survey plans that take into account the need to monitor core indicators and the availability and quality of data from other sources.• Invest in building survey analytical capacity and data archiving.
Birth and death registration	In recent decades there has been virtually no progress made in improving birth and death registration globally.Only a small minority of developing countries have a functioning system for obtaining data on births, deaths (by age and sex) and causes of death.	• Step up efforts to improve birth and death registration (including cause of death) in countries through increased coordination, technical support, and funding by relevant stakeholders.• Promote a clear strategy with tools for countries with no functioning systems.
Census	Most countries are planning to conduct a census in the 2010 round.There remain major gaps in technical support for subsequent data cleaning, analysis, projections, and dissemination.	• Promote and provide support to the 2010 census round, including data analysis, projections, dissemination.• Strengthen statistical offices' analytical capacity.
Health facility reporting systems and surveillance	Facility-based information systems continue to perform poorly in terms of data quality, timeliness, and use in decision-making.There are exceptions, and several disease-specific information systems have benefited from intensive technical quality control and financial inputs, including those for outbreak disease surveillance, eradication programs (for example against polio), tuberculosis, HIV/AIDS, and immunization coverage.	• Identify the core information needs and appropriate incentives for the improved reporting of results at local, national, and global levels, and for improved data quality and timeliness, supported by the introduction of information technology.• Support independent district and facility assessments.
Administrative systems	Data on health financing, human resources, and infrastructure are still too poor to monitor basic information on the inputs of the health system.	• Promote regular National Health Accounts (NHAs) and improved systems to monitor expenditure.• Develop comprehensive, district-based monitoring systems for service delivery and workforce.
		

The eight agencies working in global health (Box 1) agree that it is critical to strengthen the five key data sources (Table 1) and capacity for analysis, synthesis, validation, and use of health data in countries. This should enable countries to better monitor and evaluate their own progress and performance and, secondarily, allow them to respond to the increased emphasis on results and accountability [5]. The eight agencies propose four global actions to support these country goals.

**Box 1. Eight Agencies Working in Global Health**

- Bill & Melinda Gates Foundation
- GAVI
- Global Fund to Fight AIDS, Tuberculosis & Malaria
- UNAIDS
- UNFPA
- UNICEF
- World Bank
- World Health Organization

## Increase Levels and Efficiency of Investments in Health Information

There are major gaps in health information that hamper monitoring of progress towards the MDGs and other goals. Sound information is lacking to monitor trends in mortality, causes of death, morbidity, coverage of interventions, risk factors, health systems, and equity. International partners tend to be focused on indicator development and reporting requirements but need to step up their efforts to strengthen country systems including data generation to address major information gaps. Required actions include:

- Enhancing investments in country data sources and the systematic strengthening of information systems through global health partnerships and special disease initiatives as part of ongoing funding and through new efforts. A commonly used figure, by, for instance, the Global Fund to Fight AIDS, Tuberculosis and Malaria, is that 5% to 10% of program funds should be invested in data collection, monitoring, evaluation, and operational research;
- Improving the efficiency of health information investments by closer collaboration between partners in support of one strong country M&E plan that covers all major disease and health programs and all data sources.

The eight agencies commit to acting upon this goal immediately by:

- Ensuring that funding for scaling up for the MDGs and health system strengthening include systematic funding to strengthen M&E systems in countries;
- Supporting countries to develop one strong M&E plan, linked to the country health sector plan and building upon existing efforts, which forms the basis for the monitoring of global goals.

## Develop a Common Data Architecture

Information technology applications are changing the scope and modalities of data collection, transmission, storage, analysis, dissemination, and sharing. The UN organizations have invested much in, for instance, standards for data collection, such as the International Classification of Diseases and Related Health Problems (ICD), and, for data transmission, notably the Standard Data and Metadata eXchange (SDMX). But the lack of a common data architecture hampers the efficient generation and use of health information. While there can be no general blueprint, it is essential to enhance interoperability between different data systems. An explicit data architecture—describing how data are collected, stored, managed, and used, and by whom and for what purposes—is needed to ensure that the increasing diversity of actors and resources contributes evenly and sustainably to resolving the information gaps at country and global levels. The required actions include:

- Investing in developing norms and standards for all aspects of a common data architecture, which includes involvement of UN agencies, academia, and the private sector;
- Developing a global health indicator registry with standards for data, indicators, metadata, and references to analytic methods that builds upon work done in health and disease programs, promotes the implementation of the standards, and focuses on a core minimal indicator set;
- Developing and promoting interoperability standards for the health sector at both the level of individual and aggregate records.

The eight agencies commit themselves to acting upon this goal immediately by:

- Working together and enhancing investments in developing a common standard for health information, including a common indicator and metadata registry and interoperable databases.

## Strengthen Performance Monitoring and Evaluation

There is a need for more rigorous M&E of progress and performance. The IHP+ common evaluation strategic framework presents a set of principles to maximize country benefits, in line with the Paris Declaration on aid effectiveness. The general principles for large-scale public health evaluation include: collective action of all major partners; alignment with country planning and reporting cycles; balance between independence and country ownership; use of internationally accepted methods and standards; strengthening of institutional capacity and health information systems as an integral part; and appropriate and timely investment in evaluation [6].

Much more can be done to reduce the reporting burden on countries and better align the monitoring of progress towards international goals—from MDGs to other international goals—with national M&E plans that are well linked to national health sector strategic plans. Also, comparable estimates for key health indicators, such as child and maternal mortality or immunization coverage, should be made on the basis of the best possible data with the best possible methods in a comprehensible, transparent manner which allows reproduction of the estimates at country and global levels. Global technical debates are useful to improve methods and estimates but should be conducted in a manner that minimizes confusion among health planners and programmers. Required actions include:

- Improving coordination of monitoring progress in order to minimize the reporting burden on countries, supported by a common data architecture with a core set of indicators;
- Fostering methodological innovation for the collection and analysis of statistics;
- Ensuring that methods and data sources for estimates are transparent, objective, and available for sharing and review;
- Improving development of tools, software, and training programs to support country capacity building for analysis and synthesis;
- Supporting rigorous and independent evaluations of initiatives, programs, and interventions, implemented in line with the principles of the IHP+ common evaluation framework when working in countries.

The eight agencies commit to acting upon this goal immediately by:

- Ensuring that global efforts in evaluation are transparent and reproducible at the country level by investing in the development of user-friendly tools, software, and training programs in support of country capacity for analysis and synthesis.
- Investing in sound evaluation of the scaling up in a way that adheres to the principles of the common IHP+ evaluation framework, ensuring that independence and scientific rigor are balanced with country ownership and alignment with country processes.

## Increase Data Access and Use

Better access to data and statistics in the public domain could generate important benefits at country and global levels by fostering collaboration and innovation in statistical and analytic methods, both for new data collection and for better use of existing data. Examples of good practice are the Demographic and Health Surveys (DHS) [7] and, more recently, UNICEF/supported Multiple Indicator Cluster Surveys (MICS) and the International Household Survey Network at the World Bank, which archive microdata from household surveys for public access [8],[9]. Data sharing requires collaboration between primary data producers and primary and secondary users, as well as measures to protect confidentiality and security. At the country level, there is a need to enhance individual and institutional capacities for data management, including data archiving and analysis, supported by development partners and funders as an integral part of programs and projects. Required actions include:

- Enhancing country and global level access to data, statistics, and metadata in the public domain, with appropriate security and confidentiality measures;
- Developing a “code of conduct” that will facilitate the release of data into the public domain, through broad consultation among data producers, researchers, funders, government representatives, and other stakeholders, including for research microdata, large-scale surveys, and public health statistics;
- Encouraging and supporting strengthening of country capacity to use and analyze data among a wide range of stakeholders, including local statistical and research institutions.

The eight agencies commit themselves to acting upon this goal immediately by:

- Making a public commitment on behalf of each of our organizations to work with other stakeholders to develop a set of specific principles around data sharing by our organizations within two years;
- Calling upon others to do the same;
- Providing funding that enables data sharing and data management.

During this era of scaling up for better health, improved accountability and focus on results are critical to improve program implementation and reach major health goals. We call for a concerted and systematic effort by global partners, including our own agencies, to provide the impetus for support to countries in strengthening their monitoring of progress and performance, building upon what countries are doing. We also call for regular well-planned evaluation of major initiatives in a way that balances independence and scientific rigor with country ownership and alignment with country processes. The current economic slowdown corroborates the need for such investments, which can greatly increase efficiency and effectiveness. This health information agenda formulated by the agencies should be advanced further in international fora such as the International Conference on Health Information in Bangkok, Thailand, and the World Economic Forum, both in early 2010.

## Abbreviations

DHS: Demographic and Health Surveys
GAVI: Global Alliance for Vaccines and Immunisation
ICD: International Classification of Diseases and Related Health Problems
IHP+: International Health Partnership
M&E: monitoring and evaluation
MDG: Millennium Development Goal
MICS: Multiple Indicator Cluster Survey
NHA: National Health Accounts
SDMX: Standard Data and Metadata eXchange
UNAIDS: Joint United Nations Programme on HIV/AIDS
UNFPA: United Nations Population Fund
UNICEF: United Nations Children's Fund
WHO: World Health Organization
